# Camptodactyly and DiGeorge syndrome: A rare hand anomaly

**DOI:** 10.1016/j.jpra.2021.03.001

**Published:** 2021-03-19

**Authors:** C.M. Hurley, N. McHugh, S. Carr, J.L. Kelly

**Affiliations:** a Department of Plastic & Reconstructive Surgery, University Hospital Galway, Co. Galway, Ireland; b Royal College of Surgeons in Ireland, Dublin, Ireland

**Keywords:** 22q11.2, Di george, Camptodactyly, Arthrogryposis

## Abstract

The most common deletion syndrome is 22q11.2 and it effects an estimated 1 in 3000 live births. Major features of this multisystem condition include congenital abnormalities, developmental delay, learning difficulties, immunodeficiency, endocrine anomalies and an array of psychiatric disorders. However, variability in phenotype and severity may cause the diagnosis to be overlooked. Early clinical recognition and treatment of DiGeorge syndrome has been shown to increase early life survival, decrease complications and enhance overall quality of life.

Skeletal anomalies are infrequently described in 22q11.2 but a subset of patients exhibit upper and lower limb deformities. We present the case of a 5 year-old girl with bilateral fifth digit camptodactyly caused by a fibrous band, and the surgical management of this condition. The current report adds to the body of evidence that camptodactyly is a rare clinical feature of 22q11.2 deletion syndrome, and may serve as a diagnostic aid in these patients.

## Introduction

22q11.2 deletion syndrome (DS), first described in 1967, is the most common microdeletion syndrome, affecting an estimated 1 in 3000 live births.^1^ Patients present with a wide constellation of clinical features and syndromes that vary, even within families. It has an equal distribution between males and females and has a median age of diagnosis below 4 years.^1^

In infancy, DiGeorge syndrome typically presents with congenital cardiac abnormalities (ventral septal defect, tetralogy of Fallot, interrupted aortic arch), immunodeficiency, learning difficulties and hypocalcemia secondary to hypothyroidism.^2^ However, clinical features vary in severity and delayed diagnosis is common. Palatal, genitourinary, skeletal and characteristic facial features may occur. Behavioral problems and psychiatric disorders occasionally present at a later timepoint.^2^

Increased use of genome diagnostic technologies, advances in pediatric care, and a high recurrence risk of 22q11.2DS have led to a higher prevalence in a growing population.^1^^,^^2^ Early clinical recognition and treatment of DiGeorge syndrome has increased survival, reduced complications and enhanced overall quality.^2^ Due to the wide multisystem spectrum of clinical features, it may be unrecognized or underreported.^1^^,^^2^ The association with congenital hand abnormalities may facilitate early identification in newborns.

Although rare, Skeletal anomalies are described in 22q11.DS. These include Scoliosis, hypoplastic vertebrae, hemivertebrae and vertebral coronal clefts.^2^ Although extremely rare, a subset of patients exhibit upper and lower limb deformities, most notably polydactyly and clubfoot. We present a case of the rare and underreported clinical feature of camptodactyly in DiGeorge syndrome.

## Case report

**Figure 1:**
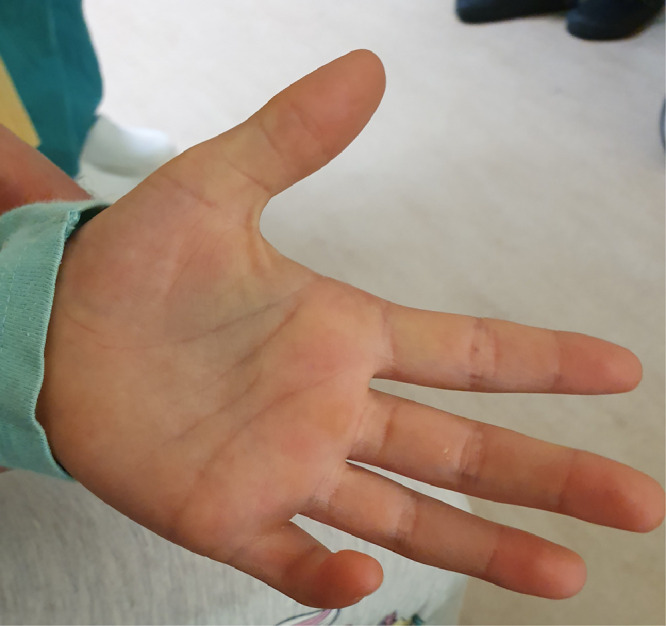
Pre-operative photograph of patients left hand in extension demonstrating little finger camptodactyly.

**Figure 2:**
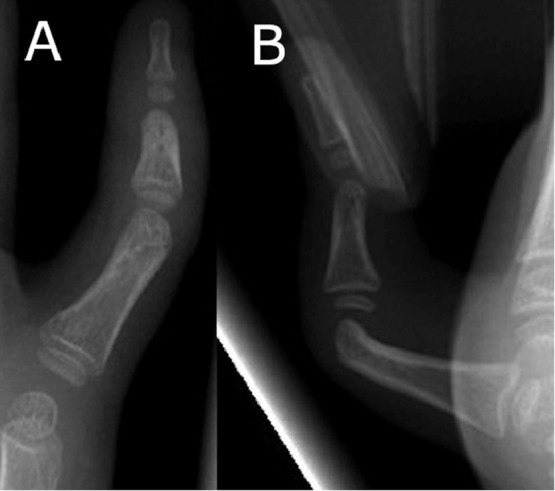
A plain x-ray identified no bony abnormality. The involved PIP joint is held in flexion (A) = Anterior posterior (B) = Lateral.

A five-year-old female presented to with bilateral progressive single digit camptodactyly. On clinical examination, she had inability to extend the distal (DIP) and proximal interphalangeal (PIP) joints of both little fingers. Passive extension of the joints was not possible and there was slight tightening of the overlying skin. At rest, her proximal interphalangeal joint was flexed to 90° (Figure 1). A non-tender tight band was palpable on the volar ulnar border of the digits. There was no obvious tethering or pitting of the subcutaneous tissues or dermis. Otherwise, the hands and feet were normal. A preoperative plain x-ray demonstrated no obvious bony abnormality (Figure 2).

The patient had a history of a ventral septal defect (VSD) and her echocardiogram demonstrated mild right cardiac hypertrophy with no other cardiac abnormality. Early learning difficulty and hypocalcemia resulted in genetic investigations at three years. An SNP microanalysis was performed, and she was subsequently diagnosed with an interstitial deletion of 22q11.2, consistent with DiGeorge syndrome. There were no other typical features of the microdeletion syndrome.

**Figure 3:**
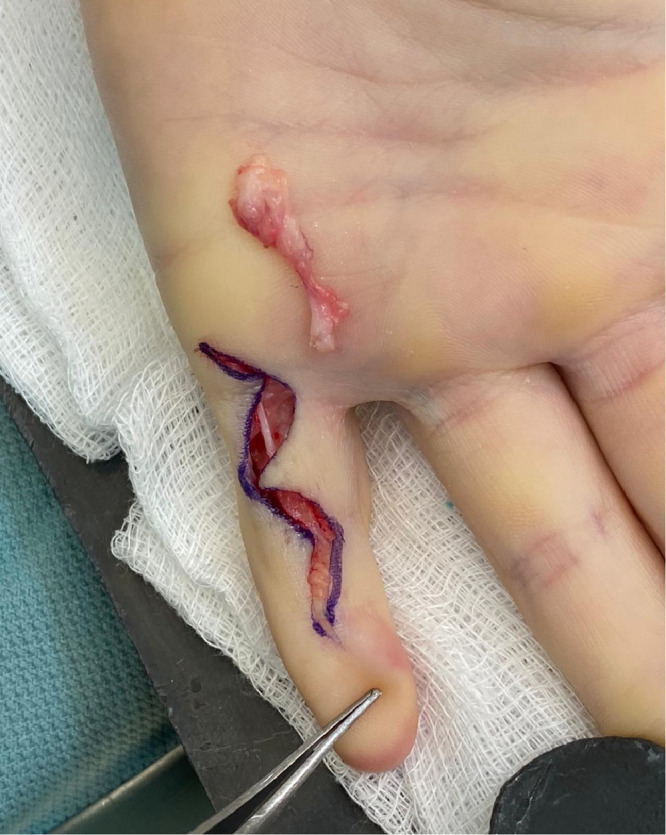
Intraoperative photograph of left little finger demonstrating Brunner's incisions and the excised band.

Surgery was performed under general anesthetic and tourniquet control. Through a Brunner's incision (Figure 3), we noted a tight band of fibrous tissue adjacent to the ulnar neurovascular bundle **(Video 1)**. This band extended from the insertion of the abductor digiti minimi to the DIP joint. It did not encase the neurovascular bundle but it prevented full extension of the PIP and DIP joints.

The tight fibroelastic band was excised (Figure 2), allowing full passive correction of both joints. The radial side of the finger was normal. No anomalous lumbrical insertion was noted. The flexor digitorum superficialis and flexor digitorum profundus were of normal size and caliber. The overlying subcutaneous tissues and dermis were healthy. As the contracture was fully corrected, we did not explore the extensor mechanism.

The patient was discharged and reviewed in a routine outpatient dressing clinic eight days later. Complete correction of the camptodactyly deformity was noted. Goniometry demonstrated 180° range of motion at the PIP and DIP joints.

The formal histopathology of the specimen demonstrated lobulated mature adipose tissue transversed by fascicles of mature fibroblasts. The proliferation demonstrated no atypia or mitotic activity and was consistent with lipofibromatosis / Dupuytren's disease.

## Discussion

Arthrogryposis is an umbrella term for several congenital joint contractures, including the inherited distal forms, amyloplastic forms, and neurogenetic conditions.^3^ Camptodactyly was first described in 1846 and is defined by fixed flexion of the PIP joint, poor active extension, intrinsic and extrinsic tendon shortening with occasional and skin deficits on the volar surface of the finger.^1^^–^^3^ The fifth finger is most often involved and can be associated with many syndromes. It presents from birth or during early childhood with various degrees of functional impairment. Surgical intervention for camptodactyly is indicated for fixed contractures that cause a functional deficit and have failed conservative management with supervised orthoses.^3^

Many causes have been described, including involvement or abnormalities of the skin, tendon sheath, flexor tendon, lumbrical and interossei, volar plate, accessory collateral ligament, and central slip insertion.^3^ Pediatric Dupuytren's Disease has been described in camptodactyly.^4^ Few reports specifically relate the condition to a syndromic phenotype. While a broad spectrum of hand abnormalities has been described for patients with 22q11.2DS, camptodactyly has only been described 10 patients.

Camptodactyly was first reported in a patient with a 22q11.2DS in 1997. Ming et al. reported three cases in a large case series of 108 patients with 22q11.2DS.^5^ Similarly, Noel et al. described three further cases in a series of 74 feto-pathological examinations.^6^ Couser et al. reported a patient with 22q11.2DS who presented with prominent bilateral multi-digit camptodactyly.^7^ This patient had multiple other phenotypical traits, including laryngeal abnormalities. Finally, Oskarsdóttir reported three further cases in a case series of 100 patients.^8^ There has been no association with Dupuytren's Disease to date.

Typically, Dupuytren's disease is a benign, progressive fibroproliferative disorder of the hand ^15^ and normally presents as nodular thickening of the palmar fascia of the metacarpophalangeal and proximal interphalangeal joints.^9^ Histologically-proven Dupuytren's disease is seldom reported in the pediatric literature.^10^ All previous pediatric cases describe a nodule in the palm or a flexion contracture of a finger.^4^ To our knowledge, this is the first report of pediatric Dupuytren's disease in a 22q11.2DS.

The malformations associated with DiGeorge syndrome have been attributed to unknown neural crest defects, including the abnormal development of organs arising from the third and fourth pharyngeal pouches.^1^ Facial defects in 22q11.2DS may be caused by defects in the first branchial arch. The association of camptodactyly with 22q11DS suggests there are polytopic developmental field defects involved in its pathogenesis.

## Conclusion

Current guidelines suggest practical strategies for the recognition, diagnosis, surveillance and management 22q11.2DS and its associated co-morbidities in both children and adults. DiGeorge syndrome constitutes an increased risk for a variety of multisystem conditions. Many patients present with manifestations that may be difficult to clinically detect, such as cardiac anomalies, immunodeficiency and mild facial features. Given the heterogeneity of signs and symptoms patients may remain undiagnosed for years. Our case adds to the body of evidence that camptodactyly is a rare clinical feature of 22q11.DS, and may serve as a clinical diagnostic aid preceding genetic testing.

## Declaration of Competing Interest

The authors of this paper have no conflict of interests to disclose.

## Appendix Supplementary materials

Video 1 Demonstration of fibrous Dupuytren's cord. Image, video 1
